# Genome-Wide Identification and Characterization of *AGO*, *DCL,* and *RDR* Gene Families in *Siraitia grosvenorii*

**DOI:** 10.3390/ijms26115301

**Published:** 2025-05-30

**Authors:** Yimei Zang, Chongnan Wang, Jiaxian Su, Changming Mo, Lei Xie, Zuliang Luo, Xiaojun Ma

**Affiliations:** 1 State Key Laboratory for Quality Ensurance and Sustainable Use of Dao-di Herbs, Institute of Medicinal Plant Development, Chinese Academy of Medical Sciences and Peking Union Medical College, Beijing 100193, China; meiyee0810@sina.com (Y.Z.); cnwang@implad.ac.cn (C.W.); jxsu@implad.ac.cn (J.S.); leixie1996@163.com (L.X.); 2 Guangxi Crop Genetic Improvement and Biotechnology Laboratory, Guangxi Academy of Agricultural Sciences, Nanning 530007, China; mochming@126.com; 3 Yuelushan Laboratory, Changsha 410006, China

**Keywords:** *Siraitia grosvenorii*, Argonaute (AGO), Dicer-like (DCL), RNA-dependent RNA polymerase (RDR)

## Abstract

RNA silencing regulates diverse cellular processes in plants. Argonaute (AGO), Dicer-like (DCL), and RNA-dependent RNA polymerase (RDR) proteins are core components of RNA interference (RNAi). Despite their functional significance, the systematic identification and characterization of these families have remained largely unexplored in *Siraitia grosvenorii*. Using HMMER and Pfam analyses, we identified six *SgAGO*, four *SgDCL*, and six *SgRDR* genes. Phylogenetic analysis classified SgAGOs, SgDCLs, and SgRDRs into five, four, and four clades, respectively, all of which clustered closely with homologs from other *Cucurbitaceae* species, demonstrating lineage-specific evolutionary conservation. Promoter *cis*-element analysis revealed the significant enrichment of hormonal (methyl jasmonate, abscisic acid) and stress-responsive (light, hypoxia) elements, indicating their roles in environmental adaptation. Tissue-specific expression profiling showed that most *SgAGO*, *SgDCL*, and *SgRDR* genes were highly expressed in flowers and mid-stage fruits (35 days after pollination), while *SgAGO10.1* exhibited stem-specific expression. By contrast, *SgRDR1.2* displayed no tissue specificity. Notably, sex-biased expression patterns in dioecious flowers suggested the RNAi-mediated regulation of gametophyte development and their potential roles in reproductive and secondary metabolic processes. This study lays the foundation for further exploration of RNAi machinery’s role in coordinating mogroside biosynthesis and stress resilience in *S. grosvenorii* while providing potential targets for genetic improvement.

## 1. Introduction

RNA silencing is a highly conserved regulatory mechanism in eukaryotes involving small non-coding RNAs that regulate gene expression at both transcriptional and post-transcriptional levels [1,2]. This mechanism plays a crucial role in modulating plant growth, development, antiviral defense, and stress responses [3]. The core components of the RNA interference (RNAi) machinery include the Argonaute (AGO), Dicer-like (DCL), and RNA-dependent RNA polymerase (RDR) protein families. These proteins collaborate to process double-stranded RNAs (dsRNAs) into small interfering RNAs (siRNAs) or microRNAs (miRNAs), which subsequently guide the RNA-induced silencing complex (RISC) to target mRNAs for cleavage or translational inhibition [2].

The AGO protein family serves as the central effector of the RISC, mediating gene silencing through guide strand binding of small RNAs (sRNAs), such as siRNAs, which leads to targeted mRNA cleavage or translational repression [4,5]. Structurally, AGO proteins are characterized by conserved functional domains, including Argo-N/Argo-L, DUF 1785, PAZ, ArgoMid, and PIWI [6]. Among these, the PAZ domain contains a critical RNA-binding site [7], while the ArgoMid domain interacts with the 5′-terminal phosphodiester bond of sRNAs [8]. The PIWI domain, which confers endonuclease activity, binds the 5′ end of siRNAs, thereby enabling RNA-guided target recognition and silencing [5].

DCL proteins, on the other hand, are ribonuclease III enzymes responsible for cleaving dsRNAs into 21–24 nucleotide sRNAs [9]. There are typically four DCL proteins in plants, each with specific roles in producing different types of sRNAs [10]. DCL proteins possess six conserved and functional domains: DEAD, Helicase-C, DUF1785, PAZ, RNase III, and DSRM. These domains are essential for the proteins to exert their functions [11,12].

RDR proteins, a major RNAi-related protein group, synthesize dsRNA using single-stranded RNA as a template. This dsRNA serves as a substrate for DCL enzymes to generate secondary siRNAs [13,14]. RDR proteins are characterized by a conserved RNA-dependent RNA polymerase (RdRP) domain [15,16,17], which is integral to their function as members of the RNA interference gene family [18].

Genome-wide studies of the AGO, DCL, and RDR families across plant species have elucidated their evolutionary conservation and functional divergence. In model plants, these families exhibit lineage-specific expansion: Arabidopsis (*Arabidopsis thaliana*) harbors 10 AGOs, 4 DCLs, and 6 RDRs [19], while rice (*Oryza sativa*) [20] and maize (*Zea mays*) [21] possess 18 AGOs, 4 DCLs, and 6 RDRs and 18 AGOs, 5 DCLs, and 11 RDRs, respectively. Members within each family display distinct spatiotemporal expression patterns and regulate diverse biological processes. Advances in genomics and computational tools have further enabled systematic analyses in non-model species, including fruit crops such as sweet orange (*Citrus sinensis*) [22], strawberry (*Fragaria* spp.) [23], banana (*Musa acuminata*) [7], grapevine (*Vitis vinifera*) [24], peach (*Prunus persica* L.) [25], pepper (*Capsicum annuum* L.) [16], and cucumber (*Cucumis sativus* L.) [26]. These investigations have delineated evolutionary trajectories, structural variations, and expression dynamics of RNAi-related genes, offering critical insights into the adaptation and diversification of RNAi machinery in horticulturally significant species.

*Siraitia grosvenorii*, commonly known as luohanguo or monk fruit, is an edible horticultural plant belonging to the family Cucurbitaceae. It is renowned for its high content of mogrosides, which are natural sweeteners with zero calories and a sweetness hundreds of times more intense than sucrose [27]. Despite its economic and medicinal importance, the *AGO*, *DCL*, and *RDR* gene families in *S. grosvenorii* remain largely uncharacterized. Identifying and characterizing these gene families in *S. grosvenorii* will provide valuable insights into RNAi machinery and its role in regulating gene expression, growth, development, and the accumulation of mogrosides in this species.

In this study, we performed a genome-wide identification and expression analysis of the *SgAGO*, *SgDCL*, and *SgRDR* gene families in *S. grosvenorii*. We employed bioinformatic approaches to search for candidate genes in the *S. grosvenorii* genome and analyzed their phylogenetic relationships, conserved domains, gene structures, and chromosomal locations. Furthermore, we investigated the transcript abundances of *SgAGO*, *SgDCL*, and *SgRDR* genes in the roots, stems, leaves, flowers, and fruits at various time points after pollination (DAP) using quantitative real-time PCR (qRT-PCR). This study provides insights into the RNAi machinery in *S. grosvenorii* and paves the way for future functional studies of these gene families in this economically and medicinally important plant species.

## 2. Results

### 2.1. Identification and Structural Analysis of SgAGO, SgDCL, and SgRDR Genes in S. grosvenorii

Using a HMMER search, we identified the putative *SgAGO*, SgDCL, and *SgRDR* genes of *S. grosvenorii*. The resulting sequences were further confirmed by analyzing conserved domains as putative family members according to the Pfam database [28]. As a result, we identified a total of six *AGO* genes, four *DCL* genes, and six *RDR* genes in *S. grosvenorii*. The detailed characteristics of all genes identified in this study, including the number of amino acids, molecular weight (kDa), theoretical pI, subcellular location, open reading frame (ORF) length, protein length (amino acid, aa), and grand average of hydropathicity (GRAVY), are listed in Table 1. The polypeptide lengths of the six identified *SgAGO* genes ranged from 927 to 1056 amino acids, with predicted molecular weights ranging from 105.33 to 117.11 kDa and theoretical pI values from 8.79 to 9.48. Four *SgDCL* genes encoded polypeptides that ranged from 1628 to 1952 amino acids in length, with molecular weights between 183.60 and 219.66 kDa, and theoretical pI values ranging from 5.84 to 8.55. Moreover, six *SgRDR* genes encoded polypeptides that ranged from 1026 to 1133 amino acids in length, with molecular weights between 116.35 and 135.98 kDa, and theoretical pI values ranging from 6.72 to 8.30.

**Figure 1:**
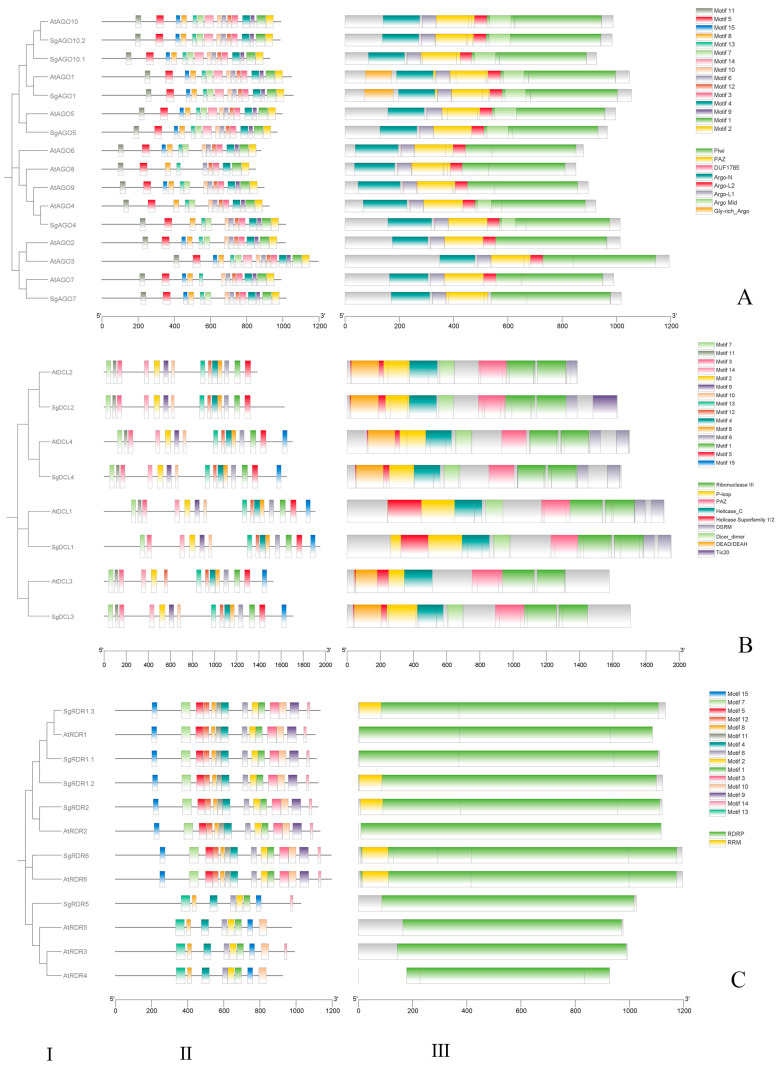
Phylogenetic relationships (I), conserved motifs (II), and conserved domains (III) of SgAGO (**A**), SgDCL (**B**), and SgRDR (**C**) families in *A. thaliana* and *S. grosvenorii*.

Conserved domain analysis via the Pfam database revealed that all SgAGO proteins shared an N-terminus PAZ domain and a C-terminus PIWI super family domain, which are the core properties of plant AGO proteins. Moreover, conserved domain analysis also showed that the ArgoL1, Argo-N, and DUF1785 domains were present in all identified SgAGO proteins. Furthermore, the Gly-rich Ago1 domain was found in SgAGO1, which is conserved in AtAGO1. Interestingly, we observed that the Argo Mid and Argo-L2 domains were present in all SgAGO variants, except SgAGO7 (Figure 1A(I,III)).

All SgDCL proteins possessed four types of conserved domains, such as Helicase_C, Helicase Superfamily 1/2, Dicer_dimer, PAZ, RNase III, and P-loop. Furthermore, SgDCL2, SgDCL3, and SgDCL 4 contained DEAD/DEAH, and a double-stranded RNA-binding domain (DSRM) was present in SgDCL1, SgDCL 2, and SgDCL 4, similar to AtDCLs. In addition, SgDCL2 contained Tic20 (Figure 1B(I,III)).

The six newly identified SgRDR proteins shared a common domain consisting of a sequence motif that corresponded to the catalytic β’ subunit of RdRP [29]. In addition, SgRDR1.2, SgRDR1.3, SgRDR2, and SgRDR6 shared another common sequence motif, the RRM domain (Figure 1C(I,III)).

### 2.2. Motif and Domain Analyses of SgAGO, SgDCL, and SgRDR in S. grosvenorii

Conserved motifs within the SgAGO, SgDCL, and SgRDR protein sequences of *S. grosvenorii* and *A. thaliana* were identified using the Multiple Em for Motif Elicitation (MEME) motif discovery tool. Comparative analysis revealed that 10 out of 20 conserved motifs were shared between AGO proteins from *A. thaliana* and *S. grosvenorii* (Figure 1A(II)). Notably, motif 15 was absent in SgAGO4, a pattern consistent with its Arabidopsis homologs AtAGO4 and AtAGO8. Furthermore, motif 3 was exclusively present in SgAGO1/5/6/10, which is also conserved in their *Arabidopsis* counterparts (AtAGO1/5/6/10), suggesting that motif 3 may represent a signature motif specific to the AGO1/5/6/10 subclade.

Within the DCL protein family, 13 out of 15 conserved motifs were universally identified across all DCL members (Figure 1B(II)). Notably, SgDCL2 lacked motif 15, a structural feature shared with its Arabidopsis homolog AtDCL2. Furthermore, SgDCL3 uniquely lacked motif 14 compared to other SgDCLs.

Within the RDR protein family, MEME analysis identified seven conserved motifs, including motifs 8, 12, 6, 9, 3, 2, 1, and 5, as major structural features shared among RDR members (Figure 1C(II)). Notably, compared to other SgRDRs, SgRDR5 lacked seven conserved motifs and harbored an additional motif, motif 13, near the N-terminus. Furthermore, motif 15 was positioned at the C-terminus of SgRDRs, a structural pattern shared with its *Arabidopsis* homologs AtRDR3/4/5.

### 2.3. Phylogenetic Analysis of SgAGO, SgDCL, and SgRDR in S. grosvenorii and Other Plant Species

**Figure 2:**
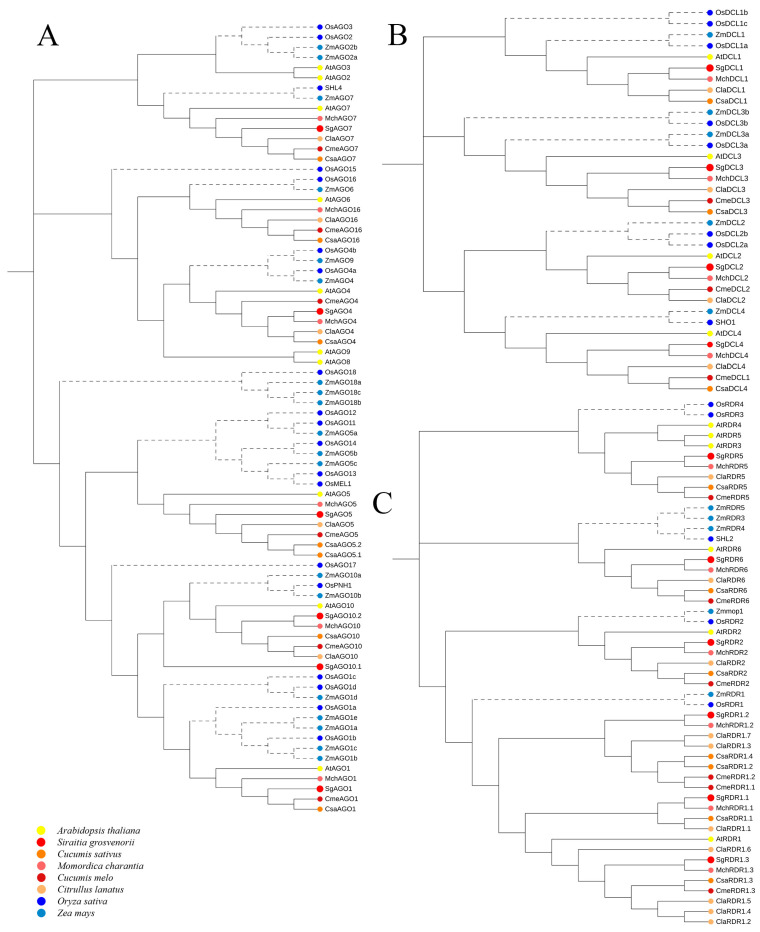
Phylogenetic analysis of the SgAGO (**A**), SgDCL (**B**), and SgRDR (**C**) proteins.

To elucidate the evolutionary relationships of SgAGO proteins, a phylogenetic tree was constructed using 78 protein sequences, which resolved into four major clades, AGO2/3/7, AGO4/6/8/9/15/16, AGO5/11/12/13/14, and AGO1/10, with an additional lineage-specific clade designated as AGO18 (Figure 2A). Each clade further diverged into monocot- and dicot-specific subclades. Notably, AGO18 formed a monocot-exclusive clade, suggesting functional divergence unique to monocots. Similarly, AGO11/12/13/14 clustered within a monocot-specific subclade yet shared ancestral affinity with the dicot AGO5 clade, indicating their origin from a common progenitor prior to monocot–dicot divergence. For the DCL family, phylogenetic analysis of 35 protein sequences delineated into four clades: DCL1, DCL2, DCL3, and DCL4 (Figure 2B). Each clade bifurcated into monocot and dicot lineages. The RDR phylogeny, comprising 49 protein sequences, resolved into four clades: RDR1, RDR2, RDR3/4/5, and RDR6 (Figure 2C). Intriguingly, ZmRDR3/4/5 and SHL2 clustered within the dicot RDR6 clade, implying a closer evolutionary relationship between these lineages.

Phylogenetic analysis revealed that SgAGO proteins clustered into four dicot-specific subclades, SgDCLs spanned all four canonical clades, and SgRDRs were assigned to four distinct clades, all demonstrating strong evolutionary conservation and close phylogenetic relationships with their homologs from other *Cucurbitaceae* species; notably, the majority of these genes formed a monophyletic branch with *M. charantia*, indicating close evolutionary relationships between *S. grosvenorii* and *M. charantia* in the SgAGO, SgDCL, and SgRDR families.

### 2.4. Protein–Protein Interactions of SgAGO, SgDCL, and SgRDR

A protein–protein interaction network for SgAGO, SgDCL, and SgRDR was constructed using the STRING 12 tool based on homologous proteins from *A. thaliana*. String mapping revealed that SgAGO, SgDCL, and SgRDR proteins aligned closely with their respective *A. thaliana* orthologs (Table 2).

**Figure 3:**
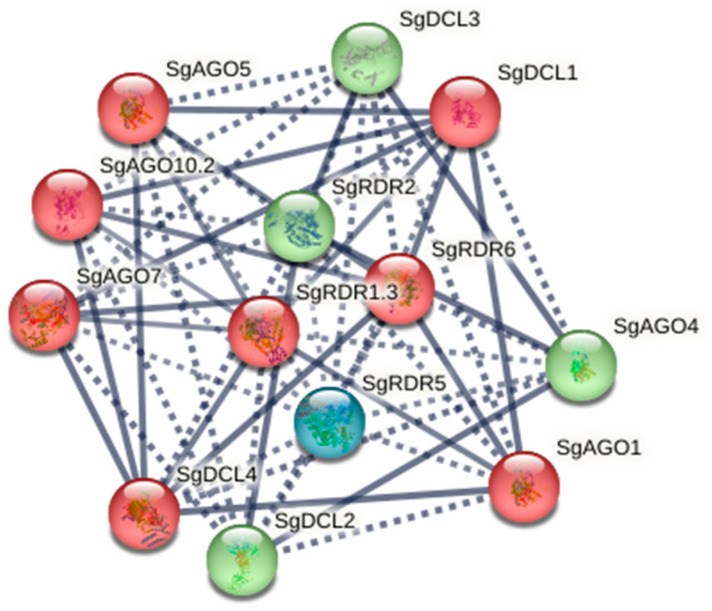
Computational prediction of the protein–protein interaction (PPI) network for SgDCLs, SgAGOs, and SgRDRs. Nodes represent proteins, colored by k-means clustering (Cluster 1: blue; Cluster 2: green; Cluster 3: red). Solid lines denote strong interactions (score > 0.7), while dotted lines indicate weaker associations (score ≤ 0.7). Proteins within the same subfamily are mapped with the highest STRING database identity scores.

Protein–protein interaction (PPI) network analysis was performed with an interaction score threshold of 0.7, and k-means clustering was employed to partition the proteins into three distinct clusters (Figure 3). Among the 16 analyzed proteins, Cluster 1 comprised SgAGO1/5/7/10, SgDCL1/4, and SgRDR1; Cluster 2 included SgAGO4, SgDCL2/3, and SgRDR2; and Cluster 3 exclusively contained SgRDR5. Strong intra-cluster interactions were observed in Clusters 1 and 2, suggesting that combinatorial associations of SgDCLs, SgAGOs, and SgRDRs may orchestrate distinct RNA silencing pathways in *S. grosvenorii*. Notably, SgRDR5 formed a solitary cluster (Cluster 3) and exhibited minimal interaction partners, implying its specialized functional role or regulatory divergence.

### 2.5. Cis-Acting Element Analysis in SgAGO, SgDCL, and SgRDR Genes

**Figure 4:**
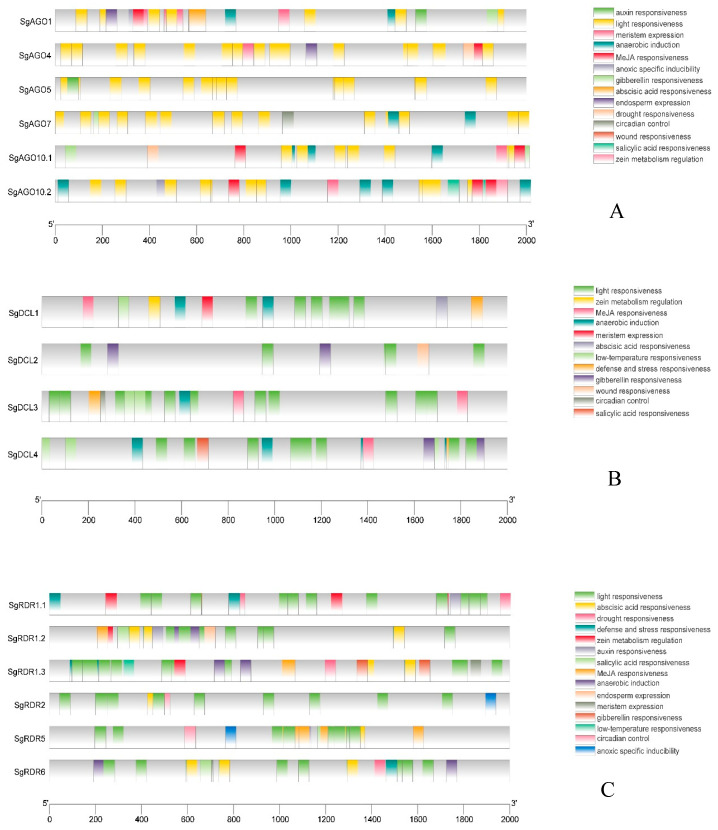
Prediction of *cis*-elements in the 2000 bp upstream regulatory regions of *SgAGO* (**A**), *SgDCL* (**B**), and *SgRDR* (**C**) genes. Different *cis*-responsive elements are represented by different colored boxes.

*Cis*-elements involved in hormone response, light response, stress response, and tissue specificity were identified in the 2000 bp upstream regulatory regions of the *SgAGO*, *SgDCL*, and *SgRDR* genes using the PlantCARE database (Figure 4, Table S5).

A total of 101 hormone-responsive elements (HREs) were identified across 375 promoter sequences of *SgDCLs*, *SgRDRs*, and *SgDCLs* in *S. grosvenorii*. Among these, 36 abscisic acid (ABA)-responsive elements were distributed as follows: 18 in *SgAGOs*, 3 in *SgDCLs*, and 15 in *SgRDRs*. Six auxin-responsive elements were detected, with two localized in *SgAGOs*, two in *SgDCLs*, and two in *SgRDRs*. For gibberellin (GA)-responsive elements, ten were identified, including four in *SgAGOs*, four in *SgDCLs*, and two in *SgRDRs*. Notably, 42 methyl jasmonate (MeJA)-responsive elements exhibited the highest abundance, with 16 in *SgAGOs*, 8 in *SgDCLs*, and 18 in *SgRDRs*. By contrast, only seven salicylic acid (SA)-responsive elements were observed, distributed as two in *SgAGOs*, one in *SgDCLs*, and four in *SgRDRs*. This distinct enrichment pattern suggested potential hormonal regulation biases in the RNAi machinery, particularly toward MeJA and ABA signaling pathways.

Additionally, a substantial number of stress-related elements (244/375) were observed, including those responsive to anaerobic or anoxic inducibility (34/244), drought (7/244), low temperature (7/244), light (186/244), defense (8/244), and wounding (2/44). Furthermore, tissue development-related and bioanabolic-responsive elements (26/375) were identified, such as those involved in endosperm expression (3/26), meristem expression (11/26), zein metabolism regulation (7/26), flavonoid biosynthesis regulation (1/26), and circadian control (4/26). Notably, the MYBHv1-binding site (4/375) was only observed on SgDCL2/3/4 promoter sequences. These results indicated that the *SgAGO*, *SgDCL*, and *SgRDR* genes may play significant roles in responding to hormones and light stress in *S. grosvenorii*.

### 2.6. Tissue-Specific Expression Patterns of SgAGO, SgDCL, and SgRDR Genes

To explore their potential roles in plant growth and development, we analyzed the expression patterns of *SgAGO*, *SgDCL*, and *SgRDR* genes across roots, stems, leaves, female flowers, and male flowers at three fruit ripening stages of *S. grosvenorii*. The spatial expression data, normalized with *S. grosvenorii ubiquitin (SgUBQ)*, were compared to the root tissue data.

**Figure 5:**
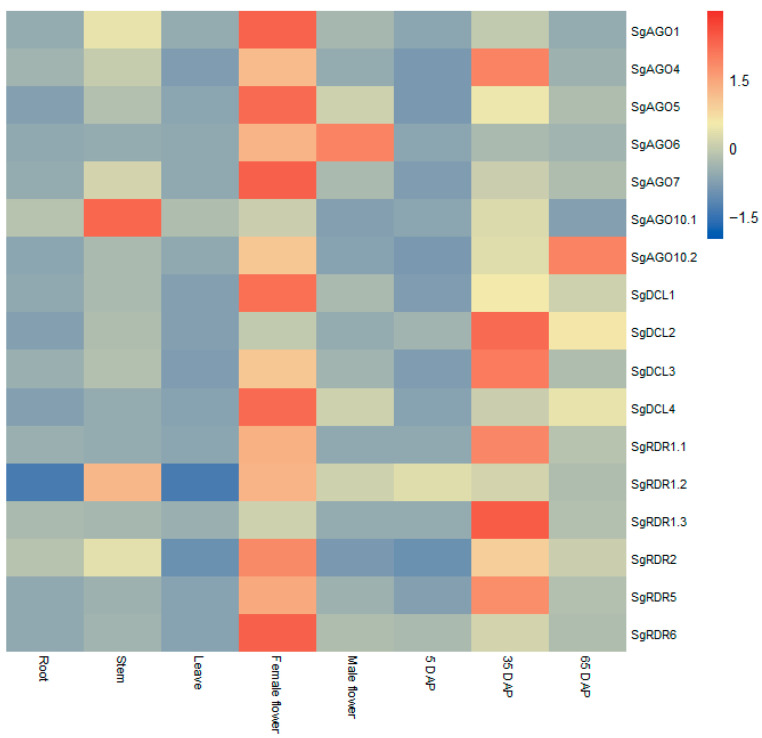
Heatmap illustrating the expression patterns of *SgAGO*, *SgDCL*, and *SgRDR* genes across various organs. Relative expression levels in *S. grosvenorii* were quantified via qRT-PCR analysis of corresponding organs such as roots, stems, leaves, female flowers, male flowers, and fruits at three ripening stages, with *SgUBQ* serving as the reference gene. The color scale representing the z-scores is displayed in the right-hand panel, with red indicating values where Z > 1.5 and blue indicating values where Z < −1.5.

Relative to root expression, *SgAGO* gene family members generally exhibited low expression in roots, leaves, and 5 DAP fruits, while showing higher expression levels in stems, female flowers, male flowers, and mid-to-late-stage fruits. Notably, *SgAGO1/5/7* displayed female flower-specific expression, with their transcript levels markedly surpassing those in other tissues. *SgAGO6* showed dual-tissue preference, peaking in male flowers followed by female flowers, both significantly higher than in other organs. *SgAGO10.1* was specifically upregulated in stems. During fruit development, *SgAGO4* reached peak expression at 35 DAP, while *SgAGO10.2* showed a unique temporal regulation pattern with maximal expression at 65 DAP. *SgDCL* gene family members were broadly repressed in leaves and 5 DAP fruits but strongly activated in female flowers and mid-to-late-stage fruits relative to their expression in root tissues. Tissue-specific divergence was observed: *SgDCL1/4* were predominantly expressed in female flowers, whereas *SgDCL2/3* peaked in 35 DAP fruits. Intriguingly, all *SgDCLs* maintained stable expression levels in stems. *SgRDR* gene family members were weakly expressed in roots and leaves but highly enriched in female flowers and mid-to-late-stage fruits as compared to roots. *SgRDR2/6* exhibited striking female flower specificity. *SgRDR1.1/1.3/5* were predominantly expressed in 35 DAP fruits. By contrast, *SgRDR1.2* displayed broad activity with minimal tissue specificity (Figure 5).

## 3. Discussion

*S. grosvenorii* (Luo Han Guo), a perennial Cucurbitaceae vine, is renowned for its abundance of nutritionally and pharmacologically valuable compounds, including fatty acids, essential amino acids, flavonoids, and triterpenoids. Of particular interest are mogrosides, a group of intensely sweet triterpenoid secondary metabolites with non-caloric properties which hold promising applications in functional foods, natural sweeteners, and traditional medicine [30]. Despite its commercial potential, *S. grosvenorii* cultivation remains restricted to narrow geographic regions, with mogroside biosynthesis, plant growth, and yield being highly sensitive to environmental fluctuations [31]. Therefore, identifying these core regulatory genes and investigating their expression patterns across different tissues and developmental stages becomes imperative. Studies in *A. thaliana* have established that the core machinery of RNAi pathways involves three conserved gene families: *AGO*, *DCL*, and *RDR* [32]. In this study, we performed genome-wide identification and functional characterization of *SgAGO*, *SgDCL*, and *SgRDR* genes in *S. grosvenorii*.

### 3.1. Evolution and Functional Divergence Mechanisms of RNAi Pathway Gene Families

In this study, through domain and phylogenetic analyses of the *SgAGO*, *SgDCL*, and *SgRDR* gene families in *S. grosvenorii*, we have preliminarily revealed the expansion patterns and conserved mechanisms of functional divergence of these gene families during evolution. Whole genome duplication (WGD) and tandem duplication are the main mechanisms driving gene family expansion. In the SgAGO family, the formation of multiple subfamilies, such as AGO2/3/7 and AGO4/6/8/9/15/16 (Figure 2A), and the monophyletic clustering of homologous genes with *M. charantia*, suggest that the ancestor of Cucurbitaceae may have undergone an ancient polyploid event. This WGD provides raw material for gene family expansion, allowing genes to explore new functions without immediately affecting organism survival [33]. Additionally, the close phylogenetic relationship between RDR1.2 and RDR1.3 in the SgRDR family (Figure 2C) and their shared RRM domain (Figure 1C(I)) indicate that tandem duplication in local chromosomal regions may have driven the expansion of this subfamily. These local duplication events often lead to gene clustering on chromosomes, promoting functional specialization within the subfamily.

After gene family expansion, domain changes become an important driving force for functional divergence. In the SgAGO family, members typically contain conserved domains such as the N-terminal domain, PAZ, and Piwi. However, SgAGO7 specifically lacks the Mid and Argo-L2 domains (Figure 1A(I)), which may result in the loss of its typical RNA-binding function. By contrast, SgAGO1–6, retaining the PAZ and PIWI domains, are likely to perform core RNAi functions. This domain difference reflects the occurrence of subfunctionalization, where different members retain partial functions of the ancestral gene during evolution, thereby achieving functional specialization. Moreover, neofunctionalization events have also emerged within the gene families. For instance, the Tic20 domain unique to SgDCL2 (Figure 1B(I)) has not been reported in other DCL homologs. This may endow SgDCL2 with unique substrate recognition capabilities, such as involvement in chloroplast RNA processing. The emergence of this new function may provide a novel selective advantage for plants to adapt to specific environmental conditions [34].

From a phylogenetic perspective, the SgAGO, SgDCL, and SgRDR families exhibit both evolutionary conservation and species-specific adaptations. SgAGO18 forms a monocot-specific clade (Figure 2A), suggesting it may have undergone unique functional divergence to meet the special physiological demands of monocots. The differentiation of SgDCL and SgRDR families between monocots and dicots (Figure 2B,C) reveals that RNAi pathway genes have balanced functional conservation with lineage-specific adaptations during plant evolution. Notably, the close clustering of SgAGO, SgDCL, and SgRDR genes with their counterparts in *M. charantia* (Figure 2) suggests that these RNAi genes may have undergone coordinated functional evolution within the family Cucurbitaceae [34]. This coordinated evolution may be related to their shared regulatory demands for secondary metabolism, potentially enhancing the adaptability of Cucurbitaceae plants.

Further domain analysis provided crucial insights into the functional diversification of SgAGO, SgDCL, and SgRDR proteins. In the SgAGO family, in addition to the aforementioned specific domain loss in SgAGO7, SgAGO1 was identified to have a glycine-rich Argo domain, a signature characteristic of AGO1 [32,35]. For DCL family proteins, all SgDCL members possess six conserved domains: Helicase_C, Helicase Superfamily 1/2, Dicer_dimer, PAZ, RNase III, and P-loop. Interestingly, SgDCL1 lacks the DEAD/DEAH domain, similar to AtDCL1 in *A. thaliana* and CsDCL1b in *C. sinensis* [35]. By contrast, SgDCL3 lacks the DSRM domain, resembling AtDCL3 in *A. thaliana* and CaDCL3 in *C. annuum* [16]. Significantly, SgDCL4 retains the PAZ domain, consistent with AtDCL4 in *A. thaliana* [36], but markedly different from CsDCL4 in *C. sinensis* [35] and HaDCL4 in sunflower (*Helianthus annuus*) [17]. Regarding the RDR protein family, all six SgRDR members harbor the conserved RdRP domain. Additionally, an RRM is present in the upstream regions of SgRDR1.2, SgRDR1.3, SgRDR2, and SgRDR6, in line with previous reports on CsRDR2 in *C. sinensis* [35] and SmRDR2 in *Salvia miltiorrhiza* [37]. These conserved and specific changes in domains collectively underpin the molecular basis for gene family functional diversification and evolution. This will enhance our comprehensive understanding of the evolution and functional mechanisms of plant RNAi pathway gene families.

### 3.2. Cis-Acting Element Analysis Reveals Hormonal and Stress Response Regulatory Mechanisms of RNAi Core Genes in S.grosvenorii

This study systematically identified *cis*-acting elements in the promoter regions of *SgAGO*, *SgDCL*, and *SgRDR* genes in *S. grosvenorii* through bioinformatics analysis and preliminarily explored their potential functions in hormonal responses and stress adaptation. The results show that the promoter regions of these three gene families contain a total of 375 *cis*-acting elements, with hormone-responsive elements accounting for 27.2% (101/375), including regulatory elements for key phytohormones such as ABA, auxin, GA, MeJA, and SA. Notably, ABA and MeJA response elements were prominently distributed in *SgAGOs* and *SgRDRs*: the *SgAGO* family was enriched with 50% ABA elements (18/36), while the *SgRDR* family carried 42.9% MeJA elements (18/42). Previous studies have revealed that *OsRDR6* in rice is positively regulated by ABA and mediates ABA-induced siRNA-dependent amplification and silencing of isocitrate lyase (ICL) transcripts [38]. Similarly, *FaRDR1k* in *Fragaria* spp. and *AcRDR1* in pineapple (*Ananas comosus*) are upregulated by ABA [23,39], whereas *FaRDR1d* and *FaRDR1g* are also activated by SA, MeJA, and GA [23]. By contrast, *SmRDRs* in *S. miltiorrhiza* are suppressed under MeJA treatment [37]. Intriguingly, previous studies demonstrated that MeJA treatment significantly upregulated the expression of key mogroside biosynthesis enzyme genes (e.g., *SgSQS*, *SgCS*, *SgCAS*, *SgCYP23/26/43*, and *SgGT1/2/4/6/7*) in *S. grosvenorii*, resulting in a 15% increase in mogroside IIE content and a 20% elevation in squalene and cucurbitadienol levels (dry weight, DW) [40], although the regulatory mechanism remains unclear. Based on these findings, we hypothesize that ABA and MeJA might indirectly influence the expression of key mogroside biosynthesis enzyme genes by regulating the amplification and silencing of *SgAGO*- or *SgRDR*-dependent siRNA transcripts, which requires further experimental validation.

Analysis of stress-responsive elements further supports the critical role of the RNAi pathway in environmental adaptation. Light-responsive elements are the most abundant (186/244) promoters of *SgAGOs*, *SgDCLs*, and *SgRDRs*, suggesting their involvement in light signal-mediated metabolism or photostress responses, consistent with the predominant distribution of light-responsive elements in *C. sinensis* [22]. Additionally, the presence of elements related to hypoxia (34/244), low temperature (7/244), and drought (7/244) provides clues for deciphering the molecular mechanisms underlying *S. grosvenorii*’s adaptation to the unique ecological environment of Guangxi mountainous regions in China. For example, *SsAGO10c*, *SsDCL2*, and *SsRDR6b* in sugarcane (*Saccharum spontaneum*) were significantly upregulated under PEG-induced dehydration stress [41], while *VvAGO1* in straw mushroom (*Volvariella volvacea*) exhibited elevated expression under cold stress, indicating its role in cold resistance [42]. Notably, the four unique MYBHv1-binding sites in the *SgDCL* promoter might participate in drought or salt stress response regulation [43,44]. These findings collectively demonstrate that the RNAi core genes in *S. grosvenorii* coordinately regulate the synthesis of secondary metabolites and adaptation to abiotic stresses by integrating hormonal signaling and stress response networks.

### 3.3. Functional Divergence of AGO, DCL, and RDR Gene Families in Tissue-Specific Expression and Developmental Regulation in S. grosvenorii

AGOs, DCLs, and RDRs function as regulators of gene silencing and modulate genomic activity in a tissue-specific manner across different developmental stages in plants. AGO proteins serve as the core components of the RISC and act as the primary effectors of the RNAi pathway [19]. This study reveals that all *SgAGO* genes are ubiquitously expressed in all tissues of *S. grosvenorii* but show differential expression levels. For instance, *AtAGO10* in *A. thaliana* [45] and *CsAGO10c* in *C. sinensis* [35] exhibit high expression in tissues with active meristem development, such as buds and stems, while *SgAGO10.1* in *S. grosvenorii* displays specific high expression in stems, suggesting its potential involvement in stem development regulation. Notably, *AtAGO5* in *A. thaliana* displays significantly higher expression during flower and seed formation compared to other tissues, and *CsAGO5a* in *C. sinensis* also shows preferential accumulation in seeds and flowers [35]. Similarly, *SgAGO5* in *S. grosvenorii* exhibits significantly higher expression in pistils, stamens, and 35 DAP fruits than in other tissues, demonstrating that *SgAGO5* might play a key role in reproductive organ development. Additionally, *AtAGO4/6/9* in *A. thaliana* mediate RNA-directed DNA methylation (RdDM) by binding with 24 nt siRNAs [46]. Recent studies demonstrated that mCHH methylation levels in *S. grosvenorii* fruits are significantly elevated during middle-to-late developmental stages compared to early stages. Furthermore, *SgAGO4/6* show markedly higher expression in reproductive organs than in vegetative tissues, suggesting that *SgAGO4/6* may function similarly to *AtAGO4/6* in de novo methylation establishment and may play a critical role in regulating reproductive organ development.

DCL family members exhibit functional redundancy in miRNA/siRNA biogenesis [47]. Although *SgDCL1/3* and *SgDCL2/4* in *S. grosvenorii* belong to distinct phylogenetic clades, their expression patterns are similar, showing significant upregulation in female flowers and middle-to-late fruit developmental stages (35 DAP). Unlike *A. thaliana* [48], *Fragaria spp.* [23], and *C. annuum* L. [16], where *DCL1/DCL3* predominantly regulate flower development, *SgDCL1/4* exhibit the highest expression in female flowers of *S. grosvenorii*, while *SgDCL3* expression is second only to that in 35 DAP fruits, suggesting collective involvement of the *SgDCL* family in flower and fruit development. Additionally, *SgDCL3* may resemble *AtDCL3* in *A. thaliana* by generating siRNAs to guide DNA methylation [46].

The RDR family is critical for dsRNA synthesis during siRNA biogenesis [1]. In *S. grosvenorii* leaves, *SgRDR1.1/1.3* exhibit extremely low expression levels. Previous studies have shown that homologous genes, such as *CmeRDR1c1/c2* in melon (*Cucumis melo*) and *CsaRDR1c1/c2* in cucumber (*C. sativus*), display negligible expression in healthy leaves but are strongly upregulated upon viral infection (e.g., cucumber mosaic virus, CMV), highlighting the essential role of *RDR1* in plant antiviral defense [49]. Except for *SgRDR1.2*, all other *SgRDRs* are highly expressed in flowers and middle-to-late fruit developmental stages (35 DAP) of *S. grosvenorii*, implying their cooperative roles in reproductive development. In *A. thaliana*, *AtRDRs* regulate female gametophyte development and fertilization processes [50,51]. Notably, the expression pattern of *SgRDR2* correlates closely with those of *SgAGO4* and *SgDCL3*, and all three are associated with the RdDM pathway, indicating that *SgRDR2* may participate in epigenetic regulation through DNA methylation mechanisms [46].

In summary, the expression of most *SgAGOs*, *SgDCLs*, and *SgRDRs* was upregulated in floral organs (particularly female flowers), with partial genes showing enhanced expression in stems. This pattern was consistent with the expression profiles of *CsaAGOs*, *CsaDCLs,* and *CsaRDRs* in *C. sativus* [26], indicating functional conservation of RNAi core genes within the family *Cucurbitaceae*. This study further found that the majority of *SgAGOs*, *SgDCLs*, and *SgRDRs* were upregulated during the 35 DAP fruit developmental stage in *S. grosvenorii*. Furthermore, as *S. grosvenorii* is a dioecious plant, differential expression of *SgAGOs*, *SgDCLs*, and *SgRDRs* was observed between female and male flowers; except for *SgAGO6*, all other genes exhibited higher expression levels in female flowers compared to male flowers. Current research on *AGO*, *DCL*, and *RDR* genes in dioecious plants remains limited. Future studies could elucidate the molecular mechanisms underlying stamen and pistil development by dissecting RNA silencing-mediated gene regulatory networks.

## 4. Materials and Methods

### 4.1. Plant Material

*S. grosvenorii* (cultivar Qingpiguo) plants were cultivated at the Yongfu County cultivation base (Guilin, China; GPS: 24°57′49.32″ N, 110°1′51.01″ E). Roots, stems, leaves, male flowers, female flowers at bloom day, and fruits at 5 DAP, 35 DAP, and 65 DAP were harvested. Three biological replicates per tissue were collected, immediately frozen in liquid nitrogen, and stored at −80 °C.

### 4.2. Data Collection and Identification of AGO, DCL, and RDR in S. grosvenorii

The protein sequences of *A. thaliana* AGO, DCL, and RDR were retrieved from the Phytozome database (https://phytozome.jgi.doe.gov/pz/portal.html, accessed on 5 December 2024) as reference sequences. The *S. grosvenorii* genome data will be published separately. *S. grosvenorii* homologs were identified using the Basic Local Alignment Search Tool (BLAST) with Hidden Markov Models (HMMs). HMMs generated position-specific scoring matrices to align query sequences against the reference database. Predicted protein sequences were extracted with >40% identity (BLOSUM62 matrix) and *E*-values < 10 × 10^−10^. To avoid redundancy, only the longest transcript per locus was retained. Identified genes were named based on phylogenetic relationships with *A. thaliana* homologs. Physicochemical properties (Mw, pI) were analyzed using ExPASy ProtParam (http://web.expasy.org/protparam, accessed on 7 December 2024). Subcellular localization was predicted via Plant-mPLoc (http://www.csbio.sjtu.edu.cn/bioinf/plant-multi/, accessed on 8 December 2024).

### 4.3. Phylogenetic Tree Construction

Protein sequences of AGO, DCL, and RDR from 14 plant species (Tables S1–S3), including model plants (*A. thaliana*, *Oryza sativa*, *Zea mays*) and Cucurbitaceae (*Cucumis sativus*, *Momordica charantia*, *Cucumis melo*, *Citrullus lanatus*), were obtained from NCBI and CuGenDBv2 (http://cucurbitgenomics.org/v2/, accessed on 5 January 2025). Multiple sequence alignment was performed using MUSCLE with default settings, specifically a Gap Opening Penalty of −2.90 and a Gap Extension Penalty of 0.00. Subsequently, maximum-likelihood trees were constructed in MEGA 11 (version 11.0.13) utilizing the Poisson correction model accompanied by gamma-distributed rate variation. Bootstrap support values were computed from 1000 replicates initiated with a random seed. The phylogenetic trees were visualized via iTOL (v6.8, https://itol.embl.de, accessed on 10 January 2025), with branch lengths adjusted according to the substitution rate and bootstrap values of ≥50% exhibited at the nodes.

### 4.4. Conserved Motif and Gene Structure Analysis

Conserved domains in SgAGO, SgDCL, and SgRDR proteins were identified via Pfam (https://pfam.xfam.org/, accessed on 15 January 2025). Conserved motifs in SgAGO, SgDCL, and SgRDR proteins were analyzed using the Multiple Em for Motif Elicitation (MEME) Suite (v5.05) program (http://meme-suite.org, accessed on 15 January 2025) [52]. Promoter regions (2000 bp upstream) of *SgAGO*, *SgDCL*, and *SgRDR* genes were screened for *cis*-acting elements using PlantCARE (http://bioinformatics.psb.ugent.be/webtools/plantcare/html/, accessed on 17 January 2025). Protein interaction networks were predicted via STRING 12 (https://string-db.org, accessed on 19 January 2025).

### 4.5. Gene Expression Analysis

Total RNA was isolated using Ve Zol Reagent (R411, Vazyme, Nanjing, China), a TRIzol-like lysis buffer, according to the manufacturer’s protocol. After RNA isolation, residual genomic DNA was digested with DNase I (EN401, Vazyme, Nanjing, China). qRT-PCR primers (Table S4) were designed in Primer Premier 6. Reactions were performed in triplicate on the CFX96™ system (Bio-Rad, Hercules, CA, USA) using SYBR Premix Ex Taq™ (Vazyme). *SgUBQ* was employed as an internal reference gene due to its stable expression across various tissues and developmental stages of fruits [53]. In qRT-PCR analysis, root tissue was designated as the basal control, and the relative expression levels of other tissues (stems, leaves, flowers, fruits) were normalized to root expression using the 2^−ΔΔCt^ method with three biological replicates. Heatmaps of relative expression levels were generated and visualized using R 4.4.2.

## 5. Conclusions

This study systematically identified and characterized the RNAi core gene families—*SgAGOs*, *SgDCLs*, and *SgRDRs*—in *S*. *grosvenorii*. Phylogenetic analysis demonstrated *Cucurbitaceae*-specific evolutionary conservation, with these genes clustering closely with homologs from related species. Promoter *cis*-element analysis revealed the enrichment of hormonal (MeJA, ABA) and stress-responsive (light, hypoxia) elements, pointing to their roles in environmental adaptation. Tissue-specific expression profiling showed predominant upregulation in flowers and mid-stage fruits (35 DAP), while *SgAGO10.1* exhibited stem-specific expression and *SgRDR2* lacked tissue specificity. Sex-biased expression patterns implicated RNAi in gametophyte development and secondary metabolism. These findings establish RNAi machinery as a critical regulator of mogroside biosynthesis and stress resilience in *S. grosvenorii*, providing foundational insights for future research.

## Figures and Tables

**Table 1:** Basic features of *SgAGO*, *SgDCL*, and *SgRDR* genes.

Gene Name	Gene Locus	Number of Amino Acids	Molecular Weight (kDa)	Theoretical pI	Grand Average of Hydropathicity (GRAVY)	Predicted Subcellular Localization
*SgAGO1*	Chr09.g17302	1056	117.11	9.31	−0.50	Nucleus
*SgAGO4*	Chr06.g10665	1014	113.60	8.79	−0.32	Cell membrane, chloroplast, and nucleus
*SgAGO5*	Chr09.g16238	967	107.54	9.48	−0.41	Nucleus
*SgAGO7*	Chr10.g18565	1018	115.58	9.37	−0.40	Chloroplast and nucleus
*SgAGO10.1*	Chr08.g15010	927	105.33	9.22	−0.39	Cell membrane and nucleus
*SgAGO10.2*	Chr03.g06089	984	110.63	9.33	−0.44	Nucleus
*SgDCL1*	Chr03.g06642	1952	219.66	5.87	−0.44	Nucleus
*SgDCL2*	Chr08.g14552	1628	183.60	8.55	−0.060	Nucleus
*SgDCL3*	Chr02.g03104	1708	191.74	6.39	−0.16	Nucleus
*SgDCL4*	Chr08.g15125	1651	186.62	6.30	−0.27	Nucleus
*SgRDR1.1*	Chr10.g17889	1112	127.79	7.89	−0.32	Chloroplast
*SgRDR1.2*	Chr01.g01240	1123	128.68	8.11	−0.38	Chloroplast and nucleus
*SgRDR1.3*	Chr10.g17890	1133	129.16	8.30	−0.29	Chloroplast
*SgRDR2*	Chr08.g15576	1121	128.81	6.72	−0.29	Nucleus
*SgRDR5*	Chr08.g14932	1026	116.35	7.89	−0.30	Chloroplast
*SgRDR6*	Chr02.g03034	1194	135.98	6.97	−0.30	Chloroplast and nucleus

**Table 2:** Shared identity between SgAGO, SgDCL, and SgRDR proteins and *A. thaliana* orthologs based on PPI network analysis.

Query Index	Query Item	Preferred Name	Identity (%)	Bitscore
1	SgAGO1	AtAGO1	82.8	1565.8
2	SgAGO4	AtAGO4	72.9	1357.8
3	SgAGO5	AtAGO5	67	1164.1
4	SgAGO7	AtAGO7	70.2	1269.6
5	SgAGO10.1	AtAGO10	74.5	1348.6
6	SgAGO10.2	AtAGO10	82.6	1661.4
7	SgDCL1	AtDCL1	71.7	2708.3
8	SgDCL2	AtDCL2	57.8	1550
9	SgDCL3	AtDCL3	47.8	1435.6
10	SgDCL4	AtDCL4	55.4	1721.8
11	SgRDR1.1	AtRDR1	61.2	1370.5
12	SgRDR1.2	AtRDR1	58.6	1266.9
13	SgRDR1.3	AtRDR1	63.7	1444.9
14	SgRDR2	AtRDR2	61.2	1403.7
15	SgRDR5	AtRDR5	51.3	866.3
16	SgRDR6	AtRDR6	67	1674.1

## Data Availability

Data is contained within the article and Supplementary Material.

## Supplementary Materials

The supporting information can be downloaded at: https://www.mdpi.com/article/10.3390/ijms26115301/s1.

## Abbreviations

The following abbreviations are used in this manuscript:

ABA	Abscisic Acid
AGO	Argonaute
DAP	Days After Pollination
DCL	Dicer-like
DSRM	Double-stranded RNA-binding domain
dsRNA	Double-stranded RNA
GA	Gibberellin
GRAVY	grand average of hydropathicity
ICL	Isocitrate lyase
MeJA	Methyl Jasmonate
MEME	Multiple Em for Motif Elicitation
ORF	open reading frame
PPI	Protein–protein interaction
qRT-PCR	Quantitative real-time PCR
RDR	RNA-dependent RNA polymerase
RdDM	RNA-directed DNA methylation
RNAi	RNA interference
RISC	RNA-induced silencing complex
SA	Salicylic Acid
sRNAs	small RNAs
siRNAs	small interfering RNAs
UBQ	Ubiquitin
